# The importance of Assertive Community Mental Health Treatment in the rural depopulation

**DOI:** 10.1192/j.eurpsy.2025.1884

**Published:** 2025-08-26

**Authors:** E. Talaya Navarro, L. Gallardo Borge, L. Martín de Francisco Murga, M. Gómez Fernández, S. Pecheykin Pajomov, M. Teijeiro Rebolo, A. Peman Alcazar

**Affiliations:** 1 Psiquiatría; 2 Complejo Asistencial de Segovia, Segovia, Spain

## Abstract

**Introduction:**

The proposed research work aims to carry out a review of the data of the Assertive Community Treatment Program of Segovia, analyzing its importance in certain profiles of psychiatric patients.

**Objectives:**

Review of data from the Segovia Assertive Community Treatment Program from 2020-2024: age, sex, nationality, marital status, level of education, diagnosis, family history, substance use, population, socio-family situation, economic situation, follow-up and admissions in Mental health, social health coordination and disability.

**Methods:**

Data from the Segovia Assertive Community Treatment Program from 2020-2024.

**Results:**

The majority of patients participating in the Segovia Assertive Community Treatment Program between 2020-2024 were Spanish men between 20 and 60 years old. All patients were single, except three separated and one married. Approximately half of them lived alone and the other half with their family of origin. Most had basic education and half were employed. Regarding the consumption of toxic substances, most of them smoked tobacco and some also consumed alcohol or cannabis, and a small number cocaine. Many of them received a financial benefit and had social-health coordination.

The most common diagnosis is schizophrenia, followed by schizoaffective disorder and delusional disorder, most with psychiatric family history. Other diagnoses that the patients presented were: bipolar disorder, personality disorder and obsessive-compulsive disorder. Some of them also had disabilities.

It is important to highlight that 94.12% of patients live in rural areas, many of them more than 30 minutes away by car and with faced great difficulties with public transportation. Many of them were more previous admissions to Psychiatry and an irregular follow-up in Mental Health, but very few had readmissions during the Program. The majority of patients had psychopharmacological treatment, and 7.14% of them had injectable antipsychotic treatment.

**Conclusions:**

A large percentage of patients in the Assertive Community Mental Health Treatment Program are people who live in a rural environment, with a long distance from the nearest mental health center and with difficulties using public transportation, which is why this program is very useful. In these patients to achieve clinical stability, since these patients had irregular follow-up in Mental Health consultations and had numerous admissions to Psychiatry.

**Disclosure of Interest:**

None Declared

